# Use of Medication for Opioid Use Disorder Among Adults With Past-Year Opioid Use Disorder in the US, 2021

**DOI:** 10.1001/jamanetworkopen.2023.27488

**Published:** 2023-08-07

**Authors:** Christopher M. Jones, Beth Han, Grant T. Baldwin, Emily B. Einstein, Wilson M. Compton

**Affiliations:** 1 National Center for Injury Prevention and Control, Centers for Disease Control and Prevention, Atlanta, Georgia; 2 National Institute on Drug Abuse, National Institutes of Health, Bethesda, Maryland

## Abstract

This cross-sectional study uses data from the 2021 National Survey on Drug Use and Health to estimate the receipt of medication for opioid use disorder among US adults with past-year opioid use disorder.

## Introduction

Many patients with opioid use disorder (OUD) do not receive medication for opioid use disorder (MOUD), including methadone, buprenorphine, or extended-release naltrexone.^1^ Data on the national prevalence of MOUD receipt among persons with OUD in the US are limited. One study using 2019 data among individuals with OUD based on *Diagnostic and Statistical Manual of Mental Disorders* (Fourth Edition) (*DSM-IV*) criteria found 27.8% received MOUD in the past year.^2^

During the COVID-19 pandemic, strategies, including expanded telehealth, were implemented to increase MOUD availability. We use data from the 2021 National Survey on Drug Use and Health (NSDUH) to provide the latest estimates of MOUD receipt among US adults with past-year OUD.

## Methods

Data were from 47 291 adults aged 18 years or older participating in the 2021 NSDUH, providing nationally representative data of the US civilian, noninstitutionalized population.^3^ This analysis focused on adults with past-year OUD based on NSDUH questions operationalizing *DSM-5* OUD diagnostic criteria. Additionally, NSDUH queries respondents’ past-year receipt of MOUD and behavioral health services, substance use, mental health, and sociodemographic characteristics (eMethods in Supplement 1). Race and ethnicity were based on self-report. NSDUH data collection was approved by RTI International’s institutional review board. Each participant provided verbal or online informed consent. Further details about NSDUH are available elsewhere.^3^ This cross-sectional study followed the STROBE reporting guideline.

Among adults with past-year OUD, we estimated prevalence of past-year substance use treatment and MOUD receipt. Multivariable logistic regression examined characteristics associated with MOUD receipt; results are presented as adjusted odds ratios (AORs) and 95% CIs, with statistical significance set at 2-sided *P* < .05. Stata, version 15 (StataCorp LP) was used for analyses, accounting for NSDUH’s complex design and sampling weights.

## Results

In 2021, an estimated 2.5 million (95% CI, 2.0-2.9 million) adults had past-year OUD. Among them, 52.5% (95% CI, 43.5%-61.4%) were men, 67.4% (95% CI, 60.1%-73.9%) were aged 35 years or older, 60.6% (95% CI, 51.8%-68.9%) were non-Hispanic White, and 52.0% (95% CI, 42.6%-61.3%) lived in large metropolitan areas.

Among adults with past-year OUD, 35.6% (95% CI, 27.6%-44.4%) received any past-year substance use treatment; 22.3% (95% CI, 15.2%-31.5%) received MOUD. Among those receiving MOUD, 58.5% (95% CI, 38.3%-76.3%) were men, 61.7% (95% CI, 43.1%-77.4%) were aged 35 years or older, 67.1% (41.3%-85.6%) were non-Hispanic White, and 57.7% (95% CI, 34.3%-78.0%) lived in large metropolitan areas.

Among adults with past-year OUD, increased odds of receiving MOUD were found among adults with severe past-year OUD (AOR, 5.45; 95% CI, 1.07-27.91) vs mild OUD, those receiving substance use treatment via telehealth in the past year (AOR, 37.78; 95% CI, 7.61-187.60), and those with family income of less than $20 000 (AOR, 6.13; 95% CI, 1.59-23.62) or $20 000 to $49 999 (AOR, 4.73; 95% CI, 1.48-15.09) vs $75 000 or more (Table). Lower odds for receiving MOUD were found among women (AOR, 0.17; 95% CI, 0.04-0.71), non-Hispanic Black adults (AOR, 0.07; 95% CI, 0.02-0.22) vs non-Hispanic White adults, those who were unemployed (AOR, 0.07; 95% CI, 0.01-0.58) vs those with full-time employment, those living in nonmetropolitan areas (AOR, 0.31; 95% CI, 0.10-0.99) vs large metropolitan areas, and those having past-year cannabis use disorder (AOR, 0.17; 95% CI, 0.04-0.76).

**Table.** Demographic and Clinical Characteristics Associated With Past-Year Receipt of Medication for Opioid Use Disorder Among Adults With Past-Year Opioid Use Disorder^a^

Characteristic	AOR (95% CI)
Sex	
Female	0.17 (0.04-0.71)^b^
Male	1 [Reference]
Age, y	
18-25	1 [Reference]
26-34	1.63 (0.28-9.43)
≥35	0.83 (0.22-3.17)
Race and ethnicity	
Hispanic	0.86 (0.22-3.35)
Non-Hispanic Black	0.07 (0.02-0.22)^b^
Non-Hispanic other^c^	2.45 (0.49-12.23)
Non-Hispanic White	1 [Reference]
Employment status	
Full-time employment	1 [Reference]
Part-time employment	0.22 (0.02-2.14)
Unemployment	0.07 (0.01-0.58)^b^
Other employment	0.16 (0.03-1.00)
Annual family income, $	
<20 000	6.13 (1.59-23.62)^b^
20 000-49 999	4.73 (1.48-15.09)^b^
50 000-74 999	3.07 (0.42-22.70)
≥75 000	1 [Reference]
Health insurance	
Medicaid	1 [Reference]
Private or other insurance	0.08 (0.01-1.01)
Uninsured	0.69 (0.18-2.66)
County of residence	
Large metropolitan area	1 [Reference]
Small metropolitan area	0.62 (0.20-1.89)
Nonmetropolitan area	0.31 (0.10-0.99)^b^
Past-year opioid use disorder severity level^d^	
Mild	1 [Reference]
Moderate	1.29 (0.25-6.73)
Severe	5.45 (1.07-27.91)^b^
Past-year co-occurring alcohol use disorder	
No	1 [Reference]
Yes	0.24 (0.05-1.11)
Past-year co-occurring cannabis use disorder	
No	1 [Reference]
Yes	0.17 (0.04-0.76)^b^
Past-year co-occurring other illicit drug use disorder^e^	
No	1 [Reference]
Yes	1.48 (0.54-4.06)
Past-year major depressive episode	
No	1 [Reference]
Yes	0.61 (0.22-1.67)
Past-year mental health treatment	
No	1 [Reference]
Yes	1.79 (0.50-6.39)
Past-year telehealth treatment for substance use	
No	1 [Reference]
Yes	37.78 (7.61-187.60)^b^

Abbreviation: AOR, adjusted odds ratio.

^a^ Among adults reporting past-year prescription opioid misuse and/or heroin misuse and meeting criteria for past-year opioid use disorder (n = 477).

^b^ Statistically significantly different from the corresponding reference group (*P* < .05).

^c^ Includes non-Hispanic Asian, non-Hispanic Native American or Alaska Native, non-Hispanic Native Hawaiian Islander or Other Pacific Islander, and non-Hispanic adults of more than 1 race.

^d^ *Diagnostic and Statistical Manual of Mental Disorders* (Fifth Edition) diagnostic criteria: mild, 2 to 3; moderate, 4 to 5; and severe, 6 or more.

^e^ Includes use disorders due to cocaine, methamphetamine, lysergic acid diethylamide, inhalants, prescription stimulants, and prescription sedatives or tranquilizers.

## Discussion

Despite guidelines recommending MOUD,^4,5^ approximately 1 in 5 adults with past-year OUD received any MOUD. Furthermore, some groups were substantially less likely to receive MOUD, in particular Black adults, women, those unemployed, and those in nonmetropolitan areas. Addressing disparities in MOUD uptake should be prioritized in program, policy, and clinical initiatives.

Consistent with prior research,^6^ receipt of telehealth treatment for substance use was associated with increased likelihood of MOUD receipt. This finding underscores the growing role telehealth can play in connecting patients with OUD to care. Limitations include NSDUH being subject to recall and social-desirability biases and lacking information about MOUD quality or duration. Findings may not generalize to groups excluded from the survey, including incarcerated individuals and people experiencing homelessness not living in shelters. Despite these limitations and the well-documented effectiveness of MOUD,^1^ our findings suggest that MOUD remains substantially underused. Future research should examine whether removal of the X-waiver in the US in 2023, along with other efforts to expand MOUD, will help close the treatment gap.

## Notes

Supplement 1. **eMethods.** National Survey on Drug Use and Health Survey Questions Related to Past-Year Opioid Use Disorder, Receipt of Substance Use Treatment, and Receipt of Medications for Opioid Use Disorder in the Past Year

Supplement 2.
Data Sharing Statement

## References

[1] National Academies of Sciences, Engineering, and Medicine. Medications for Opioid Use Disorder Save Lives. National Academies Press; 2019. 30896911

[2] Mauro PM, Gutkind S, Annunziato EM, Samples H. Use of medications for opioid use disorder among US adolescents and adults with need for opioid treatment, 2019. JAMA Netw Open. 2022;5(3):e223821. doi:10.1001/jamanetworkopen.2022.3821 35319762 PMC8943638

[3] Substance Abuse and Mental Health Services Administration. 2021 National Survey on Drug Use and Health public use file codebook. 2022. Accessed May 17, 2023. https://www.datafiles.samhsa.gov/sites/default/files/field-uploads-protected/studies/NSDUH-2021/NSDUH-2021-datasets/NSDUH-2021-DS0001/NSDUH-2021-DS0001-info/NSDUH-2021-DS0001-info-codebook.pdf

[4] American Society of Addiction Medicine. The ASAM National Practice Guideline for the treatment of opioid use disorder: 2020 focused update. 2020. Accessed May 17, 2023. https://www.asam.org/quality-care/clinical-guidelines/national-practice-guideline

[5] Substance Abuse and Mental Health Services Administration. Medications for opioid use disorder: for healthcare and addiction professionals, policymakers, patients, and families. Updated 2021. Accessed May 10, 2023. https://store.samhsa.gov/sites/default/files/pep21-02-01-002.pdf 34928548

[6] Jones CM, Shoff C, Hodges K, . Receipt of telehealth services, receipt and retention of medications for opioid use disorder, and medically treated overdose among Medicare beneficiaries before and during the COVID-19 pandemic. JAMA Psychiatry. 2022;79(10):981-992. doi:10.1001/jamapsychiatry.2022.2284 36044198 PMC9434479

