# Supplementary material for: Use of Medication for Opioid Use Disorder Among Adults With Past-Year Opioid Use Disorder in the US, 2021
Source: JAMA Netw Open. 2023 Aug 7;6(8):e2327488. doi: 10.1001/jamanetworkopen.2023.27488 (PMC10407686; doi:10.1001/jamanetworkopen.2023.27488)
Supplement: Supplement 1. — eMethods. National Survey on Drug Use and Health Survey Questions Related to Past-Year Opioid Use Disorder, Receipt of Substance Use Treatment, and Receipt of Medications for Opioid Use Disorder in the Past Year [file jamanetwopen-e2327488-s001.pdf]

Supplementary Online Content

Jones CM, Han B, Baldwin GT, Einstein EB, Compton WM. Use of medication for opioid use disorder among adults with past-year opioid use disorder in the US, 2021. *JAMA Netw Open.* 2023;6(8):e2327488. doi:10.1001/jamanetworkopen.2023.27488

eMethods. National Survey on Drug Use and Health Survey Questions Related to Past-Year Opioid Use Disorder, Receipt of Substance Use Treatment, and Receipt of Medications for Opioid Use Disorder in the Past Year

This supplementary material has been provided by the authors to give readers additional information about their work.

© 2023 Jones CM et al. *JAMA Network Open.*

eMethods. National Survey on Drug Use and Health Survey Questions Related to Past-Year Opioid Use Disorder, Receipt of Substance Use Treatment, and Receipt of Medications for Opioid Use Disorder in the Past Year

Questions Related to DSM-V Criteria for Heroin Use Disorder

DPHEINT [IF HER12MON = 1] Think about your use of **heroin** during the **past 12 months** as you answer these next questions.

Press [ENTER] to continue.

(IF HER12MON = 2, SKIP TO DPHAINT)

DPHEFEEL During the past 12 months, did you spend a **great deal of your time** using **heroin,** feeling its effects, or getting over the effects of heroin?

1 Yes
2 No
DK/REF

PROGRAMMER: SHOW 12 MONTH CALENDAR

DPHEGET [IF DPHEFEEL=2 OR DK/REF] During the past 12 months, did you spend a **great deal of your time** getting or trying to get **heroin**?

1 Yes
2 No
DK/REF

PROGRAMMER: SHOW 12 MONTH CALENDAR

DPHELRGR During the past 12 months, were there **many times** when you ended up using **heroin** in larger amounts or for a longer time than you meant to?

1 Yes
2 No
DK/REF

PROGRAMMER: SHOW 12 MONTH CALENDAR

© 2023 Jones CM et al. *JAMA Network Open.*

DPHEBDLY During the past 12 months, were there times when you wanted to use **heroin** so badly that you couldn't think of anything else?

1 Yes
2 No
DK/REF

PROGRAMMER: SHOW 12 MONTH CALENDAR

DPHEURGE [IF DPHEBDLY = 2 OR DK/REF] During the past 12 months, were there times when you had a **strong urge** to use **heroin**?

1 Yes
2 No
DK/REF

PROGRAMMER: SHOW 12 MONTH CALENDAR

DPHEMORE Do you need to use a lot more **heroin** than you used to in order to get the feeling you want?

1 Yes
2 No
DK/REF

PROGRAMMER: SHOW 12 MONTH CALENDAR

DPHELESS [IF DPHEMORE = 2 OR DK/REF] Does using the same amount of **heroin**

have much less effect on you than it used to?

1 Yes
2 No
DK/REF

PROGRAMMER: SHOW 12 MONTH CALENDAR

DPHESTOP During the past 12 months, did you **try to** cut down or **try to** stop using

heroin?

1 Yes
2 No
DK/REF

PROGRAMMER: SHOW 12 MONTH CALENDAR

© 2023 Jones CM et al. *JAMA Network Open.*

DPHECANT [IF DPHESTOP = 1] Some people who use heroin try to cut down or stop but find they can't. Was there **more than one time** in the past 12 months when you tried but were unable to cut down or stop using **heroin**?

1 Yes
2 No
DK/REF

PROGRAMMER: SHOW 12 MONTH CALENDAR

DPHEWISH [IF DPHESTOP=2 OR DK/REF] In the past 12 months, did you **often** wish that you could cut down or stop using **heroin**?

1 Yes
2 No
DK/REF

PROGRAMMER: SHOW 12 MONTH CALENDAR

DPHEPHYS During the past 12 months, did you have any long-lasting or repeated physical health problems that were caused or made worse by using **heroin**?

1 Yes
2 No
DK/REF

PROGRAMMER: SHOW 12 MONTH CALENDAR

DPHEPCNT [IF DPHEPHYS = 1]: Did you continue to use **heroin** even though it was causing long-lasting or repeated physical health problems or making your physical health problems worse?

1 Yes
2 No
DK/REF

PROGRAMMER: SHOW 12 MONTH CALENDAR

DPHEMNTL [IF DPHEPHYS = 2 OR DK/REF OR DPHEPCNT = 2 OR DK/REF]

During the past 12 months, did you have any long-lasting or repeated problems with emotions or mental health that were caused or made worse by using **heroin**?

1 Yes
2 No
DK/REF

© 2023 Jones CM et al. *JAMA Network Open.*

PROGRAMMER: SHOW 12 MONTH CALENDAR

DPHEMCNT [IF DPHEMNTL = 1]: Did you continue to use **heroin** even though it was causing long-lasting or repeated problems with your emotions or mental health or making your emotions or mental health worse?

1 Yes
2 No
DK/REF

PROGRAMMER: SHOW 12 MONTH CALENDAR

DPHEACTV This question is about important activities such as:

- Spending time with friends and family
- Attending special events at work or school
- Participating in hobbies and sports
- Attending religious services and events

During the past 12 months, did you give up or spend **a lot less time** doing any of these types of important activities because of your use of **heroin**?

1 Yes
2 No
DK/REF

PROGRAMMER: SHOW 12 MONTH CALENDAR

DPHESERI Sometimes people who use **heroin** have serious problems at work, school, or home—such as:

- missing a lot of work or school
- getting demoted, having your hours cut, or losing a job
- not being able to get a job or keep a job
- getting suspended, expelled, or dropping out of school
- failing to take care of family

During the past 12 months, did you have any serious problems like these at work, school, or home because of your use of **heroin**?

1 Yes
2 No
DK/REF

PROGRAMMER: SHOW 12 MONTH CALENDAR

© 2023 Jones CM et al. *JAMA Network Open.*

DPHEARGU During the past 12 months, did you **often** have arguments or other problems with family or friends that were caused or made worse by your use of **heroin**?

1 Yes
2 No
DK/REF

PROGRAMMER: SHOW 12 MONTH CALENDAR

DPHEACNT [IF DPHEARGU = 1]: Did you continue to use **heroin** even though it **often** caused arguments or problems with family or friends?

1 Yes
2 No
DK/REF

PROGRAMMER: SHOW 12 MONTH CALENDAR

DPHEHURT During the past 12 months, did you repeatedly get into situations where using **heroin** increased your chances of getting physically hurt?

1 Yes
2 No
DK/REF

PROGRAMMER: SHOW 12 MONTH CALENDAR

DPHEWD People may experience withdrawal symptoms when they use less or stop using **heroin**.

During the past 12 months, did you have the following withdrawal symptoms after you used less or stopped using **heroin** for a while?

	Yes	No
DPHEWD_1 Feeling kind of blue or down	◯ 1	◯ 2
DPHEWD_2 Vomiting or feeling nauseous	◯ 1	◯ 2
DPHEWD_3 Having cramps or muscle aches	◯ 1	◯ 2
DPHEWD_4 Having teary eyes or a runny nose	◯ 1	◯ 2
DPHEWD_5 Feeling sweaty, having enlarged eye pupils, or having body hair standing up on your skin	◯ 1	◯ 2

© 2023 Jones CM et al. *JAMA Network Open.*

DPHEWD_6 Having diarrhea	◯ 1	◯ 2
DPHEWD_7 Yawning	◯ 1	◯ 2
DPHEWD_8 Having a fever	◯ 1	◯ 2
DPHEWD_9 Having trouble sleeping	◯ 1	◯ 2

DK/REF

PROGRAMMER: SHOW 12 MONTH CALENDAR

DPHEOVER During the past 12 months, did you use heroin or another drug to get over or avoid having **heroin** withdrawal symptoms?

1 Yes
2 No
DK/REF

PROGRAMMER: SHOW 12 MONTH

CALENDAR PROGRAMMER:

DISPLAY IN LOWER RIGHT:

Press [F2] to see these symptoms again.

DPHEUSE [IF DPHEOVER=1] Which of the following did you use to get over or avoid having **heroin** withdrawal symptoms during the past 12 months?

	Yes	No
DPHEUSE_1 Heroin	◯ 1	◯ 2
DPHEUSE_2 Prescription pain relievers	◯ 1	◯ 2
DPHEUSE_3 Something else	◯ 1	◯ 2

DK/REF

PROGRAMMER: SHOW 12 MONTH CALENDAR

DPHEOTH [IF DPHEUSE_3=1] You indicated that you took something else to get over or avoid having **heroin** withdrawal symptoms during the past 12 months. What did you take?

____

DK/REF

DPHELAW During the past 12 months, did using **heroin** cause you to do

© 2023 Jones CM et al. *JAMA Network Open.*

things that repeatedly got you in trouble with the law?

1 Yes
2 No
DK/REF

PROGRAMMER: SHOW 12 MONTH CALENDAR

Questions Related to DSM-V Criteria for Prescription Opioid Use Disorder

DPPRINT [IF PR12MON = 1] Think about your use of **prescription pain relievers**

during the **past 12 months** as you answer these next questions.

Earlier the computer recorded that in the past 12 months you used [IF PRYRCOUNT=1 FILL PRFILL][IF PRYRCOUNT >=2 FILL WITH “the pain relievers listed below”]**.**

[IF PRYRCOUNT >=2 FILL WITH THE INDIVIDUAL DRUGS SELECTED FROM PR01-PR10 BELOW. USE MULTIPLE COLUMNS AS

NEEDED. IF PRYANYOTH = 1, ADD "some other prescription pain reliever."]

The next questions refer to [IF PRYANYOTH NE 1 AND PRYRCOUNT =1 FILL PRFILL as a prescription pain reliever; IF PRYANYOTH = 1 AND PRYRCOUNT =1 FILL WITH “this other prescription pain reliever”; IF PRYRCOUNT >=2 FILL WITH “these as prescription pain relievers”].

Press [ENTER] to continue.

(IF PR12MON = 2, SKIP TO DPTRINT)

DPPRFEEL During the past 12 months, did you spend a **great deal of your time** using any **prescription pain relievers,** feeling their effects, or getting over the effects of any prescription pain relievers?

1 Yes
2 No
DK/REF

PROGRAMMER: SHOW 12 MONTH CALENDAR

© 2023 Jones CM et al. *JAMA Network Open.*

DPPRGET [IF DPPRFEEL=2 OR DK/REF] During the past 12 months, did you spend a
great deal of your time getting or trying to get any **prescription pain relievers**?

1 Yes
2 No
DK/REF

PROGRAMMER: SHOW 12 MONTH CALENDAR

DPPRLRGR During the past 12 months, were there **many times** when you ended up using any **prescription pain relievers** in larger amounts or for a longer time than you meant to?

1 Yes
2 No
DK/REF

PROGRAMMER: SHOW 12 MONTH CALENDAR

DPPRBDLY During the past 12 months, were there times when you wanted to use any
prescription pain relievers so badly that you couldn't think of anything else?

1 Yes
2 No
DK/REF

PROGRAMMER: SHOW 12 MONTH CALENDAR

DPPRURGE [IF DPPRBDLY = 2 OR DK/REF] During the past 12 months, were there times when you had a **strong urge** to use any **prescription pain relievers**?

1 Yes
2 No
DK/REF

PROGRAMMER: SHOW 12 MONTH CALENDAR

DPPRMORE Do you need to use a lot more of any **prescription pain relievers** than you used to in order to get the feeling you want?

1 Yes
2 No
DK/REF

© 2023 Jones CM et al. *JAMA Network Open.*

PROGRAMMER: SHOW 12 MONTH CALENDAR

DPPRLESS [IF DPPRMORE = 2 OR DK/REF] Does using the same amount of any **prescription pain relievers** have much less effect on you than it used to?

1 Yes
2 No
DK/REF

PROGRAMMER: SHOW 12 MONTH CALENDAR

DPPRSTOP During the past 12 months, did you **try to** cut down or **try to** stop using any

prescription pain relievers?

1 Yes
2 No
DK/REF

PROGRAMMER: SHOW 12 MONTH CALENDAR

DPPRCANT [IF DPPRSTOP=1] Some people who use prescription pain relievers try to cut down or stop but find they can't. Was there **more than one time** in the past 12 months when you tried but were unable to cut down or stop using any **prescription pain relievers**?

1 Yes
2 No
DK/REF

PROGRAMMER: SHOW 12 MONTH CALENDAR

DPPRWISH [IF DPPRSTOP=2 OR DK/REF] In the past 12 months, did you **often** wish that you could cut down or stop using any **prescription pain relievers**?

1 Yes
2 No
DK/REF

PROGRAMMER: SHOW 12 MONTH CALENDAR

© 2023 Jones CM et al. *JAMA Network Open.*

DPPRPHYS During the past 12 months, did you have any long-lasting or repeated physical health problems that were caused or made worse by using a **prescription pain reliever**?

1 Yes
2 No
DK/REF

PROGRAMMER: SHOW 12 MONTH CALENDAR

DPPRPCNT [IF DPPRPHYS = 1]: Did you continue to use any **prescription pain relievers** even though they were causing long-lasting or repeated physical health problems or making your physical health problems worse?

1 Yes
2 No
DK/REF

PROGRAMMER: SHOW 12 MONTH CALENDAR

DPPRMNTL [IF DPPRPHYS = 2 OR DK/REF OR DPPRPCNT = 2 OR DK/REF]

During the past 12 months, did you have any long-lasting or repeated problems with emotions or mental health that were caused or made worse by using a **prescription pain reliever**?

1 Yes
2 No
DK/REF

PROGRAMMER: SHOW 12 MONTH CALENDAR

DPPRMCNT [IF DPPRMNTL = 1]: Did you continue to use any **prescription pain relievers** even though they were causing long-lasting or repeated problems with your emotions or mental health or making your emotions or mental health worse?

1 Yes
2 No
DK/REF

PROGRAMMER: SHOW 12 MONTH CALENDAR

DPPRACTV This question is about important activities such as:

- Spending time with friends and family
- Attending special events at work or school
- Participating in hobbies and sports

© 2023 Jones CM et al. *JAMA Network Open.*

- Attending religious services and events

During the past 12 months, did you give up or spend **a lot less time** doing any of these types of important activities because of your use of any **prescription pain relievers**?

1 Yes
2 No
DK/REF

PROGRAMMER: SHOW 12 MONTH CALENDAR

DPPRSERI Sometimes people who use **prescription pain relievers** have serious problems at work, school, or home—such as:

- missing a lot of work or school
- getting demoted, having your hours cut, or losing a job
- not being able to get a job or keep a job
- getting suspended, expelled, or dropping out of school
- failing to take care of family

During the past 12 months, did you have any serious problems like these at work, school, or home because of your use of any **prescription pain relievers**?

1 Yes
2 No
DK/REF

PROGRAMMER: SHOW 12 MONTH CALENDAR

DPPRARGU During the past 12 months, did you **often** have arguments or other problems with family or friends that were caused or made worse by your use of a **prescription pain reliever**?

1 Yes
2 No
DK/REF

PROGRAMMER: SHOW 12 MONTH CALENDAR

DPPRACNT [IF DPPRARGU = 1]: Did you continue to use any **prescription pain relievers** even though they **often** caused arguments or problems with family or friends?

1 Yes

© 2023 Jones CM et al. *JAMA Network Open.*

2 No
DK/REF

PROGRAMMER: SHOW 12 MONTH CALENDAR

DPPRHURT During the past 12 months, did you repeatedly get into situations where using a **prescription pain reliever** increased your chances of getting physically hurt?

1 Yes
2 No
DK/REFPROGRAMMER: SHOW 12 MONTH CALENDAR

DPPRWD People may experience withdrawal symptoms when they use less or stop using **prescription pain relievers**.

During the past 12 months, did you have the following withdrawal symptoms after you used less or stopped using any **prescription pain relievers** for a while?

	Yes	No
DPPRWD_1 Feeling kind of blue or down	◯ 1	◯ 2
DPPRWD_2 Vomiting or feeling nauseous	◯ 1	◯ 2
DPPRWD_3 Having cramps or muscle aches	◯ 1	◯ 2
DPPRWD_4 Having teary eyes or a runny nose	◯ 1	◯ 2
DPPRWD_5 Feeling sweaty, having enlarged eye pupils, or having body hair standing up on your skin	◯ 1	◯ 2
DPPRWD_6 Having diarrhea	◯ 1	◯ 2
DPPRWD_7 Yawning	◯ 1	◯ 2
DPPRWD_8 Having a fever	◯ 1	◯ 2
DPPRWD_9 Having trouble sleeping	◯ 1	◯ 2

DK/REF

PROGRAMMER: SHOW 12 MONTH CALENDAR

DPPROVER During the past 12 months, did you use any prescription pain relievers or other drugs to get over or avoid having **prescription pain reliever** withdrawal symptoms?

1 Yes
2 No

© 2023 Jones CM et al. *JAMA Network Open.*

DK/REF

PROGRAMMER: SHOW 12 MONTH

CALENDAR PROGRAMMER:

DISPLAY IN LOWER RIGHT:

Press [F2] to see these symptoms again.

DPPRUSE [IF DPPROVER=1] Which of the following did you use to get over or avoid having **prescription pain reliever** withdrawal symptoms during the past 12 months?

	Yes	No
DPPRUSE_1 Prescription pain relievers	◯ 1	◯ 2
DPPRUSE_2 Heroin	◯ 1	◯ 2
DPPRUSE_3 Something else	◯ 1	◯ 2

DK/REF

PROGRAMMER: SHOW 12 MONTH CALENDAR

DPPROTH [IF DPPRUSE_3=1] You indicated that you took something else to get over or avoid having **prescription pain reliever** withdrawal symptoms during the past 12 months. What did you take?

____

DK/REF

DPPRLAW During the past 12 months, did using **prescription pain relievers** cause you to do things that repeatedly got you in trouble with the law?

1 Yes
2 No

DK/REF

PROGRAMMER: SHOW 12 MONTH CALENDAR

Questions Related to Receipt of Substance Use Treatment Services in Past 12 Months

These next questions deal with treatment for alcohol and drug problems, **not including cigarettes**. Please report treatment or counseling designed to help you reduce or stop your alcohol or

© 2023 Jones CM et al. *JAMA Network Open.*

drug use. Please include detoxification and any other treatment for medical problems associated with your alcohol or drug use.

TX01 Have you **ever received** treatment or counseling for your use of alcohol or any drug, not counting cigarettes?

1 Yes
2 No
DK/REF

TX02 [IF TX01 = 1] During the past 12 months, that is, since [**DATEFILL**], have you received treatment or counseling for your use of alcohol or any drug, not counting cigarettes?

1 Yes
2 No
DK/REF

PROGRAMMER: SHOW 12 MONTH CALENDAR

Questions Related to Receipt of Medications for Opioid Use Disorder (MOUD) in Past 12 Months

OPMAT1 [IF TX02 = 1 AND (HE01 = 1 OR HEREF = 1 OR PRMISUSE12=1 OR

PRL01=1 OR PRL02=1)] The next question is about medication-assisted treatment prescribed by a doctor or other health professional to help reduce or stop your use of [**MATFILL]**. It is different from medications given to stop a drug overdose. Some examples of medication-assisted treatment include:

- Buprenorphine or buprenorphine-naloxone pills or film taken by mouth, also known as Suboxone, Zubsolv, Bunavail, or Subutex
- Injectable buprenorphine, also known as Sublocade
- Buprenorphine implant placed under the skin, also known as Probuphine
- Methadone
- Naltrexone pills, also known as ReVia or Trexan
- Injectable naltrexone, also known as Vivitrol

In the past 12 months, did you use medication to help reduce or stop your use of [**MATFILL]**?

1 Yes

© 2023 Jones CM et al. *JAMA Network Open.*

2 No
DK/REF

PROGRAMMER: SHOW 12 MONTH CALENDAR

OPMAT2 [IF OPMAT1=1] In the past 12 months, which of the following medications did you use to help reduce or stop your use of [**MATFILL]**?

To select more than one medication from the list, press the space bar between each number you have typed. When you have finished, press [ENTER].

1 Buprenorphine or buprenorphine-naloxone pills or film taken by muth, also known as Suboxone, Zubsolv, Bunavail, or Subutex
2 Injectable buprenorphine, also known as Sublocade
3 Buprenorphine implant placed under the skin, also known as Probuphine
4 Methadone
5 Naltrexone pills, also known as ReVia or Trexan
6 Injectable naltrexone, also known as Vivitrol
7 Some other medication to help reduce or stop your use of **[MATFILL]**

DK/REF

PROGRAMMER: SHOW 12 MONTH CALENDAR

NOTE: In the NSDUH Public Use File the only variable that is available is a Yes/No variable about receipt of MOUD in the past 12-months.

© 2023 Jones CM et al. *JAMA Network Open.*
