# Supplementary material for: Use of Medication for Opioid Use Disorder Among Adults With Past-Year Opioid Use Disorder in the US, 2021
Source: JAMA Netw Open. 2023 Aug 7;6(8):e2327488. doi: 10.1001/jamanetworkopen.2023.27488 (PMC10407686; doi:10.1001/jamanetworkopen.2023.27488)
Supplement: Supplement 2. — Data Sharing Statement [file jamanetwopen-e2327488-s002.pdf]

Data Sharing Statement

Jones. Use of Medication for Opioid Use Disorder Among Adults With Past-Year Opioid Use Disorder in the US, 2021. *JAMA Netw Open*. Published August 07, 2023. doi:10.1001/jamanetworkopen.2023.27488

Data
Data available: No

Additional Information
Explanation for why data not available: These are already publicly available data from SAMHSA.
